# Supplementary material for: Circulating Tumor Cell Subpopulations Predict Treatment Outcome in Pancreatic Ductal Adenocarcinoma (PDAC) Patients
Source: Cells. 2023 Sep 13;12(18):2266. doi: 10.3390/cells12182266 (PMC10526802; doi:10.3390/cells12182266)
Supplement: Supplementary file 1 [file cells-12-02266-s001.zip › cells-2588419-supplementary.pdf]

SUPPORTING INFORMATION

Circulating Tumor Cell Subpopulations Predict Treatment Outcome in Pancreatic Ductal Adenocarcinoma (PDAC) Patients

Ian M. Freed [1,2], Anup Kasi [3,*], Oluwadamilola Fateru [1,2], Mengjia Hu [1,2,4,5], Phasin Gonzales [1,2], Nyla Weatherington [1,2], Harsh Pathak [4,6], Stephen Hyter [4,6], Weijing Sun [3], Raed Al-Rajabi [3], Joaquina Baranda [3], Mateusz L. Hupert [7], Prabhakar Chalise [3], Andrew K. Godwin [2,4,6], Malgorzata A. Witek [1,2,4] and Steven A. Soper [1,2,4,5,7,8,9,*]

[1] Department of Chemistry, The University of Kansas, Lawrence, KS 66047, USA; ifreed@stowers.org (I.M.F.); damifat@ku.edu (O.F.); m367h922@kumc.edu (M.H.); pgonzalez@stetson.edu (P.G.); nweatherington@gmail.com (N.W.); mwitek@ku.edu (M.A.W.)

[2] Center of Bio-Modular Multiscale Systems for Precision Medicine (CBM[2]), The University of Kansas, Lawrence, KS 66047, USA; agodwin@kumc.edu

[3] Division of Medical Oncology, University of Kansas, Medical Center, Kansas City, KS 66160, USA; wsun2@kumc.edu (W.S.); ral-rajabi@kumc.edu (R.A.-R.); jbaranda@kumc.edu (J.B.); pchalise@kumc.edu (P.C.)

[4] Kansas Institute for Precision Medicine, University of Kansas Medical Center, Kansas City, KS 66160, USA; hpathak@kumc.edu (H.P.); shyter@kumc.edu (S.H.)

[5] Department of Cancer Biology, The University of Kansas Medical Center, Cancer Center, Kansas City, KS 66160, USA

[6] Department of Pathology and Laboratory Medicine, University of Kansas Medical Center, Kansas City, KS 66160, USA

[7] BioFluidica, Inc., San Diego, CA 92121, USA; matt@biofluidica.com

[8] Bioengineering Program, The University of Kansas, Lawrence, KS 66045, USA

[9] Department of Mechanical Engineering, The University of Kansas, Lawrence, KS 66045, USA

* Correspondence: akasi@kumc.edu (A.K.); ssoper@ku.edu (S.A.A.)

Reagents and chemicals.

Microfluidic chips were purchased from Biofluidica, Inc (San Diego, CA). Phosphate-buffered saline pH = 7.4 (PBS) (Gibco, Grand Island, NY), 2-(4-morpholino)-ethane sulfonic acid (MES), bovine serum albumin (BSA), Triton-X100, paraformaldehyde solution (Sigma-Aldrich, Saint Louis, MO), 1-ethyl-3-(3-dimethylaminopropyl)carbodiimide (EDC), N-hydroxysuccinimide (NHS) (Pierce, Rockford, IL), blood stabilizer eptifibatide acetate (SML1042, Sigma-Alrich), mouse anti-human EpCAM mAb (R&D Systems, clone#158210, Minneapolis, MN), mouse anti-human Fibroblast Activation Protein α (FAPα) mAb (R&D Systems, clone#427819), mouse anti-human CD4 mAb (R&D systems, clone#34930) DAPI, anti-CD45-488 mAb (R&D Systems, clone#998209), anti-Vimentin-647 mAb (R&D Systems, clone#280618) and anti-Pan-cytokeratin-570 (clone AE1/AE3, eBioscience, San Diego, CA). 1000 and 300 µL CO-RE sterile filterless pipette tips (Hamilton, Reno, NV). TRIS-glycine native running buffer 10X (Alfa Aesar, Ward Hill, MA). Trypsin, porcine pancreas (Alfa Aesar, Ward Hill, MA).

Modification of CTC Microfluidic Chips with Antibodies.

Chips surface were functionalized with UV/O_3 by the manufacturer. Antibody attachment protocol used was previously published (11). In short, attachment chemistry utilizes surface carboxylic acid groups which are activated with EDC/NHS coupling reagents, followed by direct attachment of antibodies via lysine groups located on mAb. Azide free solutions of *anti*-EpCAM, *anti*-FAPα and anti-CD4 mAbs were used at a concentration of 0.6 mg/ml in PBS pH7.4 and incubated overnight at 4°C.

Impedance detection of CTCs.

CTC enumeration was completed using an impedance detector, which has been reported on previously (11). Following CTC isolation and wash, CTCs were released from the CTC chip

surface by infusing a solution of 0.25% trypsin in 1x Tris-glycine (TG) buffer at a flow rate of 30 μL/min for 45 minutes. Trypsin partially digests the surface-bound antibodies and cell surface antigens, and releases captured CTCs from the capture chip toward the electrodes of the impedance detector. During trypsin infusion, the CTC isolation device was connected serially to the microfluidic impedance sensor using PEEK (OD 1/16") tubing and released cells were shuttled to the impedance sensor and counted. As cells that pass through the detector, they change the resistivity in the sensing zone between 75 μm diameter electrodes. Cells with intact membrane increase the resistivity of the solution, while those that have compromised membrane lower the resistivity of the sensing zone. Both types of cells generate impedance signal identified as positive or negative peaks, respectively. These events were identified as peaks in LABVIEW and enumerated using a custom MATLAB script (57).

Genomic DNA (gDNA) isolation.

Genomic DNA (gDNA) was isolated for molecular analysis such as next generation sequencing (NGS) or ligase detection reaction (LDR). Following isolation, on-chip lysis was performed using Quick gDNA™ MicroPrep kit (Zymo Research) by infusing lysing buffer through the chip and placing lysate on the column. Following manufacturers protocol, gDNA was eluted from the column using 10 μl of nuclease-free water. Collected gDNA was stored at -80 °C until further use. gDNA concentration and size was assessed using an Agilent 2200 TapeStation system (Agilent Technologies, Santa Clara, CA). For the molecular profiling between 3 and 6 ml of blood was processed through the CTC isolation chips. Also, a third device (i.e., anti-CD4 functionalized chips) was used for isolation of CD4+ T cells, to identify germline mutations. gDNA from the cell lines used in PCR/LDR assay was extracted using the Quick-gDNA ™ Microprep Kit (Zymo Research), as well.

Whole Genome Amplification (WGA).

WGA kit (Illustra Single Cell GenomiPhi DNA Amplification Kit (Cytiva, Marlborough, MA) was used to amplify gDNA extracted from CTCs to perform NGS and LDR. For WGA, a 20 μl reaction containing 3 μl of genomic DNA template (1 μl DNA (extracted from CTCs in above step), 1 μl lysis buffer, 1 μl neutralization buffer) and 17 μl of reaction mix in a 0.2 ml PCR tube was incubated at 30 °C for 2 hours followed by enzyme heat-inactivation step at 65 °C for 10 minutes. The whole genome amplified DNA was transferred to a 1.5 ml tube and purified by ethanol precipitation. For ethanol purification, a 192 μl reaction containing 20 μl of amplified DNA, 30 μl of 20 mM EDTA (Thermo Fisher Scientific, Fair Lawn, NJ), 5 μl of 3M sodium acetate (Invitrogen, Waltham, MA) and 137 μl of ice-cold 100% ethanol (Fisher Chemical, Fair Lawn, NJ) in a 1.5 ml tube was inverted several times and centrifuged at high speed (~16000 x g) for 20 minutes. Thereafter, 500 μl of ice-cold 70% ethanol was added after the supernatant was carefully discarded without disturbing the DNA pellet on the side of the tube. The tube was inverted several times and centrifuged at (~16,000 x g) for 5 minutes. The supernatant was discarded, and the DNA pellet was re-suspended in water, and incubated at 4 °C for 15 – 20 minutes while mixing gently by pipetting the solution up and down several times. The Agilent 2200 TapeStation was used to determine the length and concentration of the purified amplified gDNA samples.

Table S1. Performance metrics of the Robotic System.

Medium Processed	Set Dispensed Volume (μl)	Actual Volume Dispensed (μl)	Accuracy (%)	Set Flow Rate (μl/min)	Actual Flow Rate (μl/min)
PBS	1000 (n=8)	977 ± 3	98	1000	850
Blood	1000 (n=8)	893 ± 68	89	78	55
PBS	50 (n=2)	46	93	60	58
Lysis buffer	100 (n=26)	91 ± 6	91	25	24
PBS	100 (n=26)	93 ± 4	93	60	58
PBS	100 (n=24)	95 ± 3	95	300	225

Table S2. List of DDR genes screened for trial enrollment. Tumor tissue must be positive for at least one mutated gene; can be either germline or somatic.

BRCA1/2	*PALB2*	*ATM*	*NBN*	*ATR*
BRIP	*IDH1/2*	*RAD51*	*RAD51 B/C/D*	*RAD54L*
CDK12	*BARD1*	*FAM175A*	*BAP1*	*CHEK1/2*
GEN1	*MRE11A*	*XRCC2*	*SHFM1*	*FANCD2*
FANCA	*FANCC*	*FANCG*	*RPA1*	*ARID1A*

Table S3. Number of CTCs, non-CTCs, and WBCs isolated from blood. CTCs: DAPI(+), CK(+), Vim(+/-), CD45(-); Non-CTCs are defined as cells with phenotype: DAPI(+), VIM(+), CK(-), CD45(-); WBC are defined as cells with phenotype: DAPI(+), CD45(+), VIM(-), CK(-); no cells were present as being DAPI(+), CD45(-) and VIM(-). EOT = end of treatment. Purity = number of CTCs over total cells counted on chip. Abbreviations: n.a. (not available) means sample was not processed; n.d. - no CTCs were detected.

ID	Cycle	$CTC^{FAP\alpha}$ /ml	CTC^{EpCAM} /ml	Non-CTC/ml FAPα device	Non-CTC/ml EpCAM device	WBC/ml FAPα device	WBC/ml EpCAM device	Purity (%) FAPα device	Purity (%) EpCAM device
**020**	EOT	95.	28.	90.	21.	0.0	0.0	51.4	57.1
**023**	1	13.	35.	n.a	n.a	n.a	n.a	n.a	n.a
	2	15.	29.	6.0	2.0	0.0	0.0	71.4	93.5
	3	20.	34.	n.a	n.a	n.a	n.a	n.a	n.a
	EOT	8.0	36.	52.	57.	1.0	3.0	13.1	37.5
**024**	2	21.	50.	18.	3.0	0.0	0.0	53.8	94.3
	3	n.a	n.a	n.a	n.a	n.a	n.a	n.a	n.a
	4	65.	37.	19.	39.	1.0	2.0	76.5	47.4
	5	22.	40.	50.	90.	0.0	2.0	30.6	30.3
**025**	1	29.	44.	9.0	16.	0.0	0.0	76.3	73.3
	2	29.	41.	115	151	0.0	0.0	20.1	21.4
	5	14.	32.	16.	28.	0.0	2.0	46.7	51.6
	8	4.0	83.	8.0	7.0	0.0	0.0	33.3	92.2
	9	2.0	2.0	0.0	0.0	0.0	0.0	100	100
	10	0.5	2.0	0.0	0.5	0.0	0.5	100	66.7
	11	n.a	3.0	n.a	1.5	0.0	0.0	n.a	66.7
	12	8.0	1.0	1.5	0.0	0.0	0.0	84.2	100
	13	1.5	0.0	4.5	1.5	0.0	0.0	25.0	n.d
	14	2.0	1.0	3.0	4.0	0.0	0.0	40.0	20.0
**026**	1	11.	0.0	8.0	0.0	0.0	0.0	57.9	n.d
	2	34.	60.	23.	15.	2.0	0.0	57.6	78.9
**027**	4	65.	55.	10.	17.	0.0	0.0	86.7	76.4
**028**	1	8.0	37.	4.0	3.0	0.0	0.0	66.7	92.5
	2	13.	27.	168	240	1.0	3.0	7.10	10.0
	3	74.	20.	16.	45.	0.0	0.0	82.2	30.8
	4	1.0	10.	n.a	n.a	n.a	n.a	n.a	n.a
	5	3.0	17.	3.0	3.0	0.0	0.0	50.0	85.0
**029**	1	35.0	99.0	12.	45.	3.0	0.0	70.0	68.8
**030**	1	9.5	13.5	1.0	1.5	0.0	0.0	90.5	90.0
	3	7.0	4.0	1.5	0.5	0.0	0.0	82.4	88.9
	4	0.50	0.50	0.5	0.0	0.0	0.0	50.0	100
	5	1.5	3.5	6.5	4.5	0.0	0.0	18.8	43.8
**031**	1	0.0	0.0	0.0	0.50	0.0	0.5	n.d	n.d
	2	0.0	2.0	1.5	3.5	0.0	0.0	n.d	36.4
	3	0.0	0.0	1.5	1.0	0.0	0.0	n.d	n.d
	4	2.0	3.0	1.5	5.0	0.0	0.5	57.1	35.3
	5	0.0	1.0	1.0	0.0	0.0	0.0	n.d	100
	6	2.50	5.50	2.5	4.0	0.0	0.5	50.0	55.0
	7	0.0	1.50	0.0	2.5	0.0	0.0	n.d	37.5
	8	0.50	2.5	1.0	0.0	0.0	0.0	33.3	100
	9	2.0	1.0	0.0	1.0	0.0	0.0	100	50.0
	10	0.0	0.0	0.5	1.0	1.0	0.0	n.d	n.d
**032**	1	0.50	1.0	0.0	0.0	0.0	0.0	100	100
	2	4.0	1.5	2.0	2.5	0.5	0.5	61.5	33.3
	3	2.5	0.0	1.0	1.5	0.0	0.0	71.4	0.00
	4	2.0	1.5	0.5	1.5	0.0	0.0	80.0	50.0
	5	2.5	n.a	1.0	n.a	0.0	n.a	71.4	n.a
	6	0	2.5	1.5	3.0	0.0	0.0	n.d	45.5
	7	1.5	4.0	1.0	1.5	0.0	0.5	60.0	66.7
	8	5.5	4.0	2.5	1.5	0.0	0.0	68.8	72.7
	EOT	12	7.5	11.5	13.5	1.0	2.0	49.0	32.6
**033**	1	1.5	1.5	2.0	3.0	0.0	0.0	42.9	33.3
	2	6.0	1.5	5.0	8.5	0.0	0.5	54.5	14.3
	3	1.5	0.0	1.5	1.0	0.0	0.0	50.0	n.d
	4	6.5	21.5	2.0	9.0	0.0	0.5	76.5	69.4
	5	3.5	0.50	0.5	1.0	0.0	0.0	87.5	33.3
	6	1.0	3.0	3.0	2.5	0.5	1.0	22.2	46.2
	7	2.0	3.0	3.5	4.5	0.0	0.0	36.4	40.0
	8	4.5	6.5	7.0	2.5	0.0	0.5	39.1	68.4
	9	3.5	3.0	8.0	10.	2.0	1.5	25.9	20.7

	10	3.0	4.5	1.0	6.0	1.0	0.5	60.0	40.9
**034**	1	1.0	1.5	2.0	1.5	0.0	0.0	33.3	50.0
	2	0.0	0.5	0.0	0.0	0.0	0.0	n.d	100
	3	0.0	0.0	0.0	1.5	0.0	0.0	n.d	0.00
	4	13.5	2.5	8.5	6.0	1.0	0.0	58.7	29.4
	5	4.5	4.0	1.5	2.0	0.5	0.5	69.2	61.5
	6	5.5	2.5	3.0	0.0	1.0	0.0	57.9	100
	EOT	6.5	3.5	3.0	2.5	0.5	0.5	65.0	53.8
**035**	1	4.0	2.5	1.0	5.0	0.0	0.0	80.0	33.3
	2	n.a	n.a	n.a	n.a	n.a	n.a	n.a	n.a
	3	4.5	2.0	5.0	2.7	0.5	1.0	45.0	33.3
	4	1.0	0.0	0.0	0.0	0.0	0.0	100	n.d
	5	2.0	1.5	2.0	1.5	0.0	0.0	50.0	60.0
	6	8.5	4.5	3.0	4.5	1.0	0.0	68.0	50.0
	7	6.5	9.0	5.0	3.5	0.0	0.0	56.5	72.0

Table S4. Number of CTCs isolated per ml whole blood for each sample using the impedance detector.

Pt ID	Cycle	$CTC^{FAP\alpha}$/ ml	CTC^{EpCAM} /ml	Blood volume (ml)
**001**	1	29.	43.5	2
	2	5.5	3.0	2
**002**	1	109.5	72.0	2
	2	23.5	34.5	2
	-	5.5	5.5	2
	EOT	11.5	8.0	2
**003**	1	47.	36.	2
	2	67.5	27.	2
	EOT	11.5	2.0	2
**004**	1	80.	150	2
	2	3.5	7.5	2
	3	28.5	15.	2
**005**	1	25.	41.	2
	2	10.5	12.5	2
**006**	1	25.	3.5	2
	3	21.5	4.0	2
	4	17.5	13.	2
	5	9.0	21.	2
	6	25.5	69.	2
	7	5.0	10.5	2
	8	5.5	1.0	2
	EOT	29.5	13.5	2
**008**	1	0.50	1.0	2
	3	19.5	0.50	2
**009**	1	20.5	3.0	2
	3	9.0	40.	2
**010**	1	0.0	8.5	2
**011**	1	11.0	8.0	2
	2	19.0	5.5	2
**013**	1	1.5	0.50	2
	2	55.	15.5	2
	3	3.0	35.5	2
**014**	2	44.	49.	2
	4	4.5	6.0	2
	5	3.0	6.0	2
	6	0.50	17.	2
	EOT	18.	3.0	2
**015**	1	1.5	6.5	2
	2	45.	38.	2
**016**	1	14.5	33.	2
	2	29.	15.5	2
	3	74.	18.	2
	5	8.5	0.50	2
**101-016**	1	7.0	5.5	2
	EOT	0.50	0.50	2
**793**	1	14.5	8.0	2
**020**	1	68.5	81.	2
	4	1.0	19.	2
	5	15.5	5.0	2

	6	24.	41.	1
**809**	2	29.	2.0	2

Table S5. Averages and ranges of CTCs detected in PDAC patients' blood. Not all patients were able to complete all treatment cycles, therefore, number of patients in second column varies. "EOT" in the table is "End of Treatment" and is the last blood sample received from a patient (n=22). Clinical sensitivity % defines percentage of samples in which the number of CTCs isolated when threshold specificity was set at 100%, based on assay performed with healthy donors' blood (n=11) (see **Table S6**).

Treatment Cycle #	Number of Patients	Average CTC/2 ml (CTC range/ 2 ml)		Clinical Sensitivity (%)		
		$CTC^{FAP\alpha}$	CTC^{EpCAM}	$CTC^{FAP\alpha}$	CTC^{EpCAM}	$CTC^{FAP\alpha}$ and CTC^{EpCAM}
**1**	27	42.0 (0.0 – 219)	54.5 (0.0 – 300)	92.6	88.9	96.3
**2**	21	43.2 (0.0 -135)	40.3 (1.0 – 120)	90.5	90.5	95.2
**3**	16	35.3 (0.0 – 148)	22.6 (0.0 – 80)	87.5	68.8	87.5
**4**	13	29.4 (1.0 – 130)	27.2 (0.0 – 110)	100	84.6	100
**5**	14	13.9 (0.0 – 44.0)	25.8 (1.0 – 80)	92.9	92.3	100
**6**	8	16.9 (0.0 – 51.0)	36.3 (5.0 – 138)	87.5	100	100
**7**	8	33.6 (0.0 – 190)	15.6 (3.0 – 56)	87.5	100	100
**8**	5	8.0 (1.0 – 11)	38.8 (2.0 – 166)	100	100	100
**9**	5	19.6 (4.0 – 59.0)	10.8 (2.0 – 27.0)	100	100	100
**EOT**	22	35.0 (0.0 – 190)	32.5 (0.0 – 198)	93.1	86.2	96.6

Table S6. Healthy blood sample counts for different cell phenotypes isolated. CTC phenotype: DAPI(+), CK(+), VIM(+/-), CD45(-). Non-CTC phenotype: DAPI(+), VIM(+), CK(-), CD45(-). WBC phenotype: DAPI(+), CK(-), CD45(+), VIM(-). 2 ml blood per device was processed.

Cell Counts following immunophenotyping of healthy controls						
**Sample ID**	**CTC phenotype FAPα device (per 2 ml)**	**CTC phenotype EpCAM device (per 2 ml)**	**Non-CTC phenotype FAPα device (per 2 ml)**	**Non-CTC phenotype EpCAM device (per 2 ml)**	**WBC phenotype FAPα device (per 2 ml)**	**WBC phenotype EpCAM device (per 2 ml)**
**HB-1**	0	0	1.0	3.0	0	0
**HB-2**	0	0	0	4.0	0	1.0
**HB-3**	0	0	3.0	5.0	1.0	1.0
**HB-4**	0	0	17.	5.0	2.0	0
**HB-5**	0	0	6.0	35.	0	3.0
**HB-6**	0	0	9.0	9.0	1.0	1.0
**HB-7**	0	0	7.0	13.	0	2.0
**HB-8**	0	3	10.	15.	1.0	0
**HB-9**	1	0	11.	7.0	0	0
**HB-10**	0	0	6.0	10.	0	1.0
**HB-11**	2	1	7.0	9.0	1.0	1.0
**Avg**	0.27	0.36	7.0	10.	0.55	0.90
**StDev**	0.65	0.92	4.8	8.9	0.69	0.94

Table S7. Number of CTCs in each processed sample that expressed VIM in addition to CK. Abbreviations: n.a. (not available) means sample was unable to be properly processed; n.d. (not determinable) means data point calculation missing key value (e.g., sample processed properly, however no CTCs were detected).

Pt ID	Cycle	Vol Blood (ml)	Total no. $CTC^{FAP\alpha}$	Total no. CTC^{EpCAM}	Total no. $CTC^{FAP\alpha}$ expressing VIM	Total no. CTC^{EpCAM} expressing VIM	% VIM Expression $CTC^{FAP\alpha}$	% VIM Expression CTC^{EpCAM}
**020**	7	1	95	28	90	21	94.7	75.0
**023**	1	1	13	35	n.a	n.a	n.a	n.a
	2	1	15	29	15	22	100	75.9
	3	1	20	34	17	22	85.0	64.7
	4	1	8	36	8	30	100	83.3

**024**	2	1	21	50	20	50	95.2	100
	4	1	66	37	53	37	81.5	100
	5	1	22	40	17	33	77.3	82.5
**025**	1	1	29	44	27	32	93.1	72.7
	2	1	29	41	26	34	89.7	82.9
	5	1	14	32	12	21	85.7	65.6
	8	1	4	83	3	40	75.0	48.2
	9	1	2	2	2	2	100	100
	10	2	1	4	1	4	100	100
	11	2	n.a	6	n.a	5	n.a	83.3
	12	2	16	2	14	2	87.5	100
	13	2	3	0	3	n.d	100	n.a
	14	2	4	2	3	2	75.0	100
**026**	1	1	11	0	9	n.d	81.8	n.a
	2	1	34	60	29	40	85.3	66.7
**027**	4	1	65	55	60	44	92.3	80.0
**028**	1	1	8	37	7	23	87.5	62.2
	2	1	13	27	10	20	76.9	74.1
	3	1	74	20	69	19	93.2	95.0
	4	1	1	10	1	7	100	70.0
	5	1	3	17	3	11	100	64.7
**029**	1	1	35	99	35	98	100	99.0
**030**	1	2	19	27	16	24	84.2	88.9
	3	2	14	8	12	5	85.7	62.5
	4	2	1	1	1	1	100	100
	5	2	3	7	3	6	100	85.7
**031**	1	2	0	0	n.d	n.d	n.d	n.d
	2	2	0	4	n.d	3	n.d	75.0
	3	2	0	0	n.d	n.d	n.d	n.d
	4	2	4	6	4	6	100	100
	5	2	0	2	n.a	1	n.a	50.0
	6	2	5	11	5	8	100	72.7
	7	2	0	3	0	1	n.d	33.3
	8	2	1	5	1	3	100	60.0
	9	2	4	2	1	0	25.0	0.0
	10	2	0	0	n.d	n.d	n.d	n.d
**032**	1	2	1	2	1	1	100	50.0
	2	2	8	3	8	2	100	66.7
	3	2	5	0	5	n.d	100	n.d
	4	2	4	3	4	1	100	33.3
	5	2	5	n.a	5	n.a	100	n.a
	6	2	0	5	n.d	1	n.d	20.0
	7	2	3	8	3	5	100	62.5
	8	2	11	8	9	7	81.8	87.5
	9	2	24	15	21	14	87.5	93.3
**033**	1	2	3	3	3	2	100	66.7
	2	2	12	3	8	3	66.7	100
	3	2	3	0	3	n.d	100	n.a
	4	2	13	43	10	18	76.9	41.9
	5	2	7	1	6	1	85.7	100
	6	2	2	6	2	3	100	50.0
	7	2	4	6	3	5	75.0	83.3
	8	2	9	13	9	10	100	76.9
	9	2	7	6	4	5	57.1	83.3
	10	2	6	9	6	2	100	22.2
**034**	1	2	2	3	2	0	100	0.00
	2	2	0	1	n.d	1	n.d	100
	3	2	0	0	n.d	n.d	n.d	n.d
	4	2	27	5	18	2	66.7	40.0
	5	2	9	8	7	4	77.8	50.0
	6	2	11	5	11	1	100	20.0
	7	2	13	7	13	5	100	71.4
**035**	1	2	8	5	5	5	62.5	100
	2	2	n.a	n.a	n.a	n.a	n.a	n.a
	3	2	9	4	8	3	88.9	75.0
	4	2	2	0	2	0	100	n.a
	5	2	4	3	4	2	100	66.7
	6	2	17	9	13	7	76.5	77.8
	7	2	13	18	8	11	61.5	61.1

CTC %VIM expression	$CTC^{FAP\alpha}$ (n=63 samples)	CTC^{EpCAM} (n=62 samples)
**Avg**	**89.1**	**69.1**
**StDev**	**14.4**	**26.8**

Table S8. Summary of patient information including CT results. Patients had CT scans prior to treatment start, as part of the enrollment process; therefore, some patients have progression/stable disease observation made *via* CT at cycle 1. Baseline CT scan date provided in "Cycle" column.

Pt ID	Survival Status	Age	Sex	Treatment Start	Treatment End	Cycle	Scan Results
001	Deceased	52	F	02/22/2019	04/28/2019	02/08/2019<br>1<br>2	Baseline<br>n.a<br>Progression
002	Deceased	75	F	02/25/2019	12/12/2019	02/01/2019	Baseline

						1	Stable
						2	n.a
						3	Progression
						EOT	Progression
003	Deceased	74	M	03/13/2019	08/16/2019	03/10/2019	Baseline
						1	Progression
						2	n.a
						EOT	Progression
004	Deceased	51	M	03/15/2019	05/28/2019	03/08/2019	Baseline
						1	Progression
						2	n.a
						3	n.a
005	Deceased	60	M	04/03/2019	07/16/2019	04/01/2019	Baseline
						1	Progression
						2	n.a
006	Deceased	67	F	04/19/2019	11/06/2021	04/17/2019	Baseline
						1	Progression
						3	Progression
						4	n.a
						5	Stable
						6	n.a
						7	Stable
						8	n.a
						EOT	Progression
008	Deceased	52	M	05/21/2019	01/29/2020	04/29/2019	Baseline
						1	n.a
						3	Progression
009	Deceased	52	F	06/12/2019	09/12/2019	05/29/2019	Baseline
						1	Progression
						3	Progression
010	Deceased	58	M	06/19/2019	08/10/2019	06/17/2019	Baseline
						1	Progression
011	Deceased	75	F	06/17/2019	09/06/2019	05/24/2019	Baseline
						1	n.a
						2	n.a
013	Deceased	52	M	07/01/2019	03/14/2020	06/18/2019	Baseline
						1	Stable
						2	n.a
						3	Progression
014	Deceased	60	M	08/05/2019	02/12/2020	07/31/2019	Baseline
						2	Stable
						4	n.a
						5	Stable
						6	n.a
						EOT	Progression
015	Alive	54	M	08/19/2019	07/07/2020	08/13/2019	Baseline
						1	Progression
						2	Progression
016	Deceased	73	M	11/27/2019	05/19/2020	11/26/2019	Baseline
						1	Progression
						2	n.a
						3	Progression
						5	Progression
101-016	Deceased	58	M	10/02/2019	12/14/2019	09/25/2019	Baseline
						1	Progression
						EOT	Progression
793	Deceased	76	M	-	08/16/2021	01/28/2020	Baseline
						1	Stable
020	Deceased	54	F	03/17/2020	01/01/2021	03/11/2020	Baseline
						1	Stable
						4	n.a
						5	Stable
						6	n.a
						EOT	Progression
809	Deceased	76	F	-	-	06/07/2020	Baseline
						2	Stable
023	Deceased	43	M	08/17/2020	04/16/2021	08/13/2020	Baseline
						1	Progression
						2	Stable
						3	Progression
						EOT	n.a
024	Deceased	62	M	09/08/2020	10/12/2021	08/19/2020	Baseline
						2	n.a
						3	Stable
						4	n.a
						5	Progression
025	Deceased	82	M	10/28/2020	01/04/2022	09/27/2020	Baseline
						1	Stable
						2	Stable
						5	Progression
						8	Progression
						9	n.a
						10	Progression
						11	n.a

						12	Progression
						13	n.a
						14	Progression
026	Alive	61	M	12/15/2020	03/02/2022	11/30/2020	Baseline
						1	Progression
						2	n.a
027	Deceased	72	F	02/02/2021	01/22/2022	01/13/2021	Baseline
						4	n.a
028	Deceased	65	M	03/05/2021	08/22/2021	03/03/2021	Baseline
						1	n.a
						2	n.a
						3	n.a
						4	Progression
						5	Progression
029	n.a	n.a	n.a	05/03/2021	n.a	n.a	n.a
030	Deceased	68	F	06/22/2021	01/18/2022	06/14/2021	Baseline
						1	Stable
						3	Stable
						4	Progression
						5	Progression
031	Alive	78	F	08/24/2021	06/03/2022	08/20/2021	Baseline
						1	Stable
						2	n.a
						3	Stable
						4	Stable
						5	Stable
						6	n.a
						7	Progression
						8	Progression
						9	n.a
						10	Progression
032	Alive	53	M	10/15/2021	06/08/2022	10/14/2021	Baseline
						1	Stable
						2	n.a
						3	Stable
						4	n.a
						5	Stable
						6	n.a
						7	Stable
						8	Stable
						EOT	n.a
033	Alive	83	F	11/10/2021	Still enrolled	11/09/2021	Baseline
						1	n.a
						2	Stable
						3	n.a
						4	n.a
						5	n.a
						6	Stable
						7	n.a
						8	Stable
						9	n.a
						10	Stable
034	Deceased	73	M	11/15/2021	05/07/2022	10/29/2021	Baseline
						1	n.a
						2	Stable
						3	n.a
						4	n.a
						5	n.a
						6	n.a
						EOT	Progression
035	Alive	48	M	02/23/2022	Still enrolled	02/18/2022	Baseline
						1	Stable
						2	n.a
						3	Stable
						4	n.a
						5	Stable
						6	n.a
						7	Stable

Table S9. Φ and CA19-9 levels for each patient by cycle number. EOT in cycle is "end of treatment" meaning the patient concluded treatment (i.e., no response to treatment or patient's death). Abbreviations: n.a. (not available) means sample was not processed or collected; n.d. (not determined) meant that CTC not detected to determine Φ. CA19-9 levels marked with (‡) mean that it was immeasurable; either being too high (>17,000 U/ml) or too low (<37 U/ml). Cycle #s with (**) mean patient still enrolled in therapy at time of data cut-off.

Pt ID	Cycle #	Analysis time (day)	Φ	CA19-9 (U/ml)

001	1	0	0.67	2772
	2(EOT)	28	1.83	9915
002	1	0	1.52	272
	2	28	0.68	382
	3	63	1.00	635
	4(EOT)	123	1.44	1752
003	1	0	1.31	2338
	2	28	2.50	2104
	3(EOT)	49	5.75	n.a
004	1	0	0.53	>17000(‡)
	2	28	0.47	>17000(‡)
	3(EOT)	55	1.90	>17000(‡)
005	1	0	0.61	>17000(‡)
	2(EOT)	26	0.84	>17000(‡)
006	1	0	7.14	1503
	3	57	5.38	1094
	4	85	1.35	905
	5	113	0.43	698
	6	141	0.37	1065
	7	176	0.48	1644
	8	204	5.50	2037
	9(EOT)	239	2.19	3805
008	1	0	0.50	22
	3(EOT)	64	39.0	11
009	1	0	6.83	>17000(‡)
	3(EOT)	61	0.23	>17000(‡)
010	1	0	n.d	>17000(‡)
011	1	0	1.38	8706
	2(EOT)	29	3.45	11374
013	1	0	3.00	303
	2	27	3.55	612
	3(EOT)	57	0.08	859
014	2	30	0.90	220
	4	54	0.75	163
	5	79	0.50	288
	6	110	0.03	694
	7(EOT)	140	6.00	1320
015	1	0	0.23	1448
	2(EOT)	30	1.18	1360
016	1	0	0.44	1633
	2	28	1.87	1238
	3	57	4.11	1032
	5(EOT)	112	17.00	1417
101-016	1	0	1.27	16689
793	1	0	1.81	2001
020	1	0	0.85	664
	4	82	0.05	167
	5	112	3.10	236
	6	152	0.59	1778
	7(EOT)	182	3.39	4086
809	2	0	14.50	1109
023	1	0	0.37	1109
	2	26	0.52	200
	3	56	0.59	629
	5(EOT)	81	0.22	n.a
024	2	30	0.42	76
	3	59	n.a	160
	4	86	1.76	238
	5(EOT)	111	0.55	348
025	1	0	0.66	2(‡)
	2	34	0.71	<1(‡)
	5	141	0.44	1(‡)
	8	225	0.05	2(‡)
	9	253	1.00	2(‡)
	10	281	0.25	2(‡)
	11	309	n.a	3(‡)
	12	337	8.00	3(‡)
	13	368	3.00	2(‡)
	14(EOT)	399	2.00	1(‡)(
026	1	0	11.0	413
	2(EOT)	26	0.57	338
027	4	104	1.18	<1(‡)
028	1	0	0.22	<1(‡)
	2	28	0.48	<1(‡)
	3	60	3.70	<1(‡)
	4	102	0.10	<1(‡)
	5(EOT)	109	0.18	0
029	1	0	0.35	102
030	1	0	0.70	102
	3	54	1.75	271
	4	79	1.00	511

	5(EOT)	105	0.43	1474
031	1	0	n.d	102
	2	16	n.d	74
	3	44	n.d	55
	4	72	0.67	44
	5	100	n.d	37
	6	142	0.45	29
	7	170	n.d	36
	8	228	0.20	38
	9	255	2.00	38
	10(EOT)	282	n.d	33
032	1	0	0.50	413
	2	28	2.67	363
	3	63	5.00	155
	4	93	1.33	250
	5	121	n.a	509
	6	157	n.d	342
	7	178	0.38	536
	8	212	1.38	1571
	9(EOT)	233	1.60	2121
033	1	0	1.00	1(‡)
	2	28	4.00	<1(‡)
	3	62	2.00	<1(‡)
	4	110	0.30	2(‡)
	5	138	7.00	4(‡)
	6	166	0.33	2(‡)
	7	196	0.67	3(‡)
	8	222	0.69	2(‡)
	9	250	1.17	3(‡)
	10**	271	0.67	4(‡)
034	1	0	0.67	40
	2	28	n.d	17
	3	56	n.d	19
	4	91	5.40	17
	5	119	1.13	39
	6	147	2.20	69
	7(EOT)	173	1.86	127
035	1	0	1.60	4779
	2	28	n.a	1781
	3	56	2.25	1585
	4	84	2.00	970
	5	112	1.33	986
	6	140	1.89	993
	7**	168	0.72	715

Table S10. Summary of the data for whole genome amplified genomic (WGA) gDNA isolated from CTCs and CD4$^+$ T-cells. WGA was purified and suspended in 10 µl of nuclease free water.

Pt ID	Cycle #	WGA DNA size range (bp)			Concentration of WGA gDNA (ng/µl)		
		CTC^{FAPα}	CTC^{EpCAM}	CD4$^+$ T cells	CTC^{FAPα}	CTC^{EpCAM}	CD4$^+$ T cells
002	C1D1	401 – 48552	398 – 51443	401 – 53203	55.5	45.3	48.2
	EOT	393 – 48510	399 – 50581	400 – 53055	12.6	10.6	20.6
005	C1D1	402 – 48656	401 – 47667	n.a	77.9	102	n.d
006	C1D1	400 – 51188	403 – 52010	397 – 54541	40.9	56.5	52.3
	EOT	406 – 50752	399 – 50752	392 – 50581	31.5	38.7	20.1
016	C1D1	392 – 52511	401 – 48888	n.a	87.4	39.2	n.d
	EOT	392 – 52076	390 – 59092	n.a	48.2	49.0	n.d
020	C1D1	398 – 49241	398 – 47711	397 – 47057	206	7.60	134
	EOT	396 – 49612	392 – 49929	400 – 50207	306	212	224
025	C1D1	400 – 52754	400 – 49790	400 – 47999	286	379	433
	C8D1	395 – 49680	399 – 53443	n.a	101	74.0	n.d
	C10D1	394 – 49202	402 – 49859	394 – 43866	77.9	68.6	4.78
028	C3D1	402 – 48233	398 – 48233	402 – 49181	86.8	67.7	140
029	C1D1	398 – 48549	402 – 47110	n.a	24.0	100	n.a
033	C4D1	401 – 49322	397 – 50124	n.a	210	524	n.a

Table S11. CTC NGS results for patient #2. Colors of gene match colors of donut on **Fig. 4B**.

								Start of Treatment			End of Treatment		
	Chr	Gene	Type	Ref.	Allele	Coding region change in longest transcript	Region	Coverage	Freq.	quality	Coverage	Freq	Avg quality
CTC^{EpCAM}	2	BARD1		C	G	NM_000465.3:c.1603G>C	215617245	0	0	n.a	14205	99.2	46.2
	3	FANCD2	SNV	G	A	NM_033084.4:c.1924G>A	10105572	1491	4.4	55.0	0	0	n.a
	8	NBN	SNV	G	T	NM_001024688.2:c.17C>A	90993660	1971	5.1	49.6	0	0	n.a
	11	ATM	SNV	A	T	NM_000051.3:c.762A>T	108115614	0	0	n.a	580	4.5	47.5
	13	BRCA2	Ins	-	A	NM_000059.3:c.1813dupA	32907420^32907421	5342	36.3	50.9	0	0	n.a
	13	BRCA2	Ins	-	A	NM_000059.3:c.9253dupA	32954272^32954273	2038	7.0	50.2	0	0	n.a
	15	IDH2	Ins	-	C	NM_002168.3:c.435dupG	90631917^90631918	3669	4.8	49.2	0	0	n.a
	17	CDK12	Del	TCC	-	NM_016507.3:c.3770_3772delCTC	37686861..37686863	6055	37.1	49.4	261	98.5	50.4
	17	CDK12	Ins	-	G	NM_016507.3:c.4382dupG	37687471^37687472	3272	11.3	46.1	0	0	n.a
	17	BRCA1	SNV	C	T	NM_007300.3:c.863G>A	41246685	0	0	n.a	1445	4.1	51.9
$CTC^{FAP\alpha}$	2	BARD1		C	G	NM_000465.3:c.1603G>C	215617245	0	0	n.a	26013	99.3	49.4
	4	FAM175A	SNV	C	T	NM_139076.2:c.391G>A	84391441	2581	5.5	42.1	0	0	n.a
	4	FAM175A	SNV	G	T	NM_139076.2:c.387C>A	84391445	2535	5.2	41.6	0	0	n.a
	8	NBN	SNV	T	A	NM_001024688.2:c.1400A>T	90965671	0	0	n.a	320	10.9	48.0
	13	BRCA2	Ins	-	A	NM_000059.3:c.1813dupA	32907420^32907421	3776	36.3	47.7	0	0	n.a
	15	IDH2	Ins	-	C	NM_002168.3:c.435dupG	90631917^90631918	4838	5.5	46.2	0	0	n.a
	17	CDK12	Del	TCC	-	NM_016507.3:c.3770_3772delCTC	37686861..37686863	4853	47.3	46.6	108	97.2	43.0
	17	CDK12	Ins	-	G	NM_016507.3:c.4382dupG	37687471^37687472	2567	9.4	43.8	0	0	n.a
	17	BRIP1	SNV	A	T	NM_032043.2:c.3681T>A	59760726	0	0	n.a	541	4.6	41.1

Table S12. CD4 T-Cell NGS results for patient #2. Colors of gene match colors of donut on Fig. 4B.

								Start of Treatment			End of Treatment		
	Chr	Gene	Type	Ref.	Allele	Coding region change in longest transcript	Region	Coverage	Freq.	quality	Coverage	Freq	Avg quality
CD4+ T Cells	2	BARD1		C	G	NM_000465.3:c.1603G>C	215617245	0	0	n.a	15733	99.5	49.1
	2	BARD1	SNV	T	A	NM_000465.3:c.1787A>T	215610469	0	0	n.a	270	14.8	52.3
	3	ATR	Ins	-	T	NM_001184.3:c.7260dupA	142178157^142178158	1414	4.4	51.6	0	0	n.a
	11	MRE11	Ins	-	T	NM_001330347.1:c.1dupA	94225966^94225967	512	4.9	52.4	0	0	n.a
	11	ATM	SNV	G	A	NM_000051.3:c.2269G>A	108128226	0	0	n.a	1083	13.9	48.9
	11	ATM	SNV	A	T	NM_000051.3:c.4300A>T	108160392	0	0	n.a	502	4.2	36.7
	11	ATM	SNV	T	A	NM_000051.3:c.4297T>A	108160389	0	0	n.a	495	4.0	38.4
	13	BRCA2	Ins	-	A	NM_000059.3:c.1813dupA	32907420^32907421	2370	28.4	50.5	0	0	n.a
	15	IDH2	Ins	-	C	NM_002168.3:c.435dupG	90631917^90631918	6053	6.5	48.3	0	0	n.a
	17	CDK12		-	C	NM_016507.3:c.3871dupC	37686961^37686962	1128	5.8	51.2	0	0	n.a
	17	CDK12	Ins	-	G	NM_016507.3:c.4382dupG	37687471^37687472	1315	7.9	44.9	820	5.7	51.1
	17	CDK12	Del	TCC	-	NM_016507.3:c.3770_3772delCTC	37686861..37686863	2601	36.2	48.6	1242	2.2	52.6
	17	BRIP1	SNV	T	C	NM_032043.2:c.1259A>G	59876542	0	0	n.a	302	5.6	38.5

Table S13. CTC NGS results for patient #6. Colors of gene match colors of donut on Fig. 4B.

								Start of Treatment			End of Treatment		
	Chr	Gene	Type	Ref.	Allele	Coding region change in longest transcript	Region	Coverage	Freq	Avg quality	Coverage	Freq	Avg quality
CTC^{EpCAM}	2	GEN1	Ins	-	A	NM_001130009.2:c.130dupA	17941333^17941334	4791	0.8	50.9	716	5.3	54.1
	2	BARD1	SNV	C	G	NM_000465.3:c.1603G>C	215617245	0	0	n.a	28580	98.0	48.8
	3	FANCD2	SNV	T	G	NM_033084.4:c.1156T>G	10088285	4856	7.6	49.9	0	0	n.a
	3	ATR	Ins	-	A	NM_001184.3:c.7457dupT	142177845^142177846	0	0	n.a	882	21.0	51.2
	3	ATR	SNV	C	T	NM_001184.3:c.525G>A	142281719	0	0	n.a	647	5.1	50.8
	13	BRCA2	Ins	-	A	NM_000059.3:c.7177dupA	32929161^32929162	2792	1.0	48.6	773	9.1	51.9
	13	BRCA2	SNV	C	T	NM_000059.3:c.8048C>T	32937387	0	0	n.a	518	45.6	50.6
	13	BRCA2	SNV	C	G	NM_000059.3:c.8165C>G	32937504	1381	46.3	48.7	0	0	n.a
	15	IDH2	Ins	-	C	NM_002168.3:c.435dupG	90631917^90631918	6555	4.8	47.5	1347	4.7	51.0
	16	FANCA	SNV	T	G	NM_001286167.1:c.236A>C	89880975	0	0	n.a	5807	6.8	51.4
	16	FANCA	MNV	CA	TT	NM_001286167.1:c.238_239delinsAA	89880972..89880973	0	0	n.a	5835	6.5	51.9
	17	CDK12	Ins	-	G	NM_016507.3:c.4382dupG	37687471^37687472	3260	6.3	46.0	334	11.4	46.7
	17	RAD51L3-RFFL, RAD51D	SNV	T	C	NM_001142571.1:c.269A>G	33443932	1791	35.6	47.4	320	27.2	51.5
	22	CHEK2	SNV	T	C	NM_001005735.1:c.1222A>G	29092891	0	0	n.a	750	4.9	55.0
$CTC^{FAP\alpha}$	2	GEN1	Ins	-	A	NM_001130009.2:c.771dupA	17952517^17952518	943	6.5	52.6	0	0	n.a
	2	BARD1	SNV	G	A	NM_000465.3:c.1690C>T	215610566	715	5.0	50.2	0	0	n.a
	2	BARD1	SNV	C	G	NM_000465.3:c.1603G>C	215617245	0	0	n.a	12602	98.7	46.3
	3	FANCD2	SNV	T	G	NM_033084.4:c.1156T>G	10088285	1527	13.2	53.6	0	0	n.a
	3	ATR	Ins	-	T	NM_001184.3:c.6618dupA	142186844^142186845	0	0	n.a	289	8.0	53.7
	3	ATR	Ins	-	T	NM_001184.3:c.5440dupA	142217556^142217557	1880	8.8	51.5	0	0	n.a
	4	FAM175A	SNV	G	A	NM_139076.2:c.1066C>T	84383786	0	0	n.a	479	9.6	53.8
	4	FAM175A	SNV	A	C	NM_139076.2:c.404T>G	84391428	0	0	n.a	554	6.1	45.2
	8	NBN	Ins	-	T	NM_001024688.2:c.1150dupA	90967511^90967512	0	0	n.a	476	6.1	54.7
	11	ATM	SNV	G	T	NM_000051.3:c.355G>T	108106420	943	13.1	50.3	0	0	n.a
	11	ATM, C11orf65	Ins	-	A	NM_000051.3:c.8432dupA	108216476^108216477	396	7.3	50.6	0	0	n.a
	13	BRCA2	SNV	C	G	NM_000059.3:c.8165C>G	32937504	703	34.1	51.1	336	99.1	49.5
	14	RAD51B	Del	AG	-	NM_001321814.1:c.502_503delGA	68352634..68352635	0	0	n.a	1281	6.6	52.5
	15	IDH2	Ins	-	C	NM_002168.3:c.435dupG	90631917^90631918	2112	5.6	53.3	1418	4.4	52.9
	17	RAD51L3-RFFL, RAD51D	SNV	T	C	NM_001142571.1:c.269A>G	33443932	759	18.4	50.6	0	0	n.a
	17	CDK12	SNV	C	T	NM_016507.3:c.1948C>T	37646826	0	0	n.a	440	6.1	52.3
	17	CDK12	SNV	G	A	NM_016507.3:c.2135G>A	37649030	605	4.5	30.3	0	0	n.a
	17	CDK12	SNV	G	A	NM_016507.3:c.2138G>A	37649033	625	4.3	33.0	0	0	n.a
	17	CDK12	Ins	-	G	NM_016507.3:c.4382dupG	37687471^37687472	939	9.5	50.6	0	0	n.a
	17	CDK12	Ins	-	C	NM_016507.3:c.3871dupC	37686961^37686962	864	8.9	49.9	0	0	n.a
	17	BRCA1	Ins	-	T	NM_007300.3:c.668dupA	41247864^41247865	924	4.0	55.4	0	0	n.a
	22	CHEK2	SNV	G	A	NM_001005735.1:c.1556C>T	29090054	0	0	n.a	369	15.4	45.0
	22	CHEK2	Ins	-	A	NM_001005735.1:c.1005dupT	29099524^29099525	2077	7.2	54.6	0	0	n.a

Table S14. CD4 T-Cell NGS results for patient #6. Colors of gene match colors of donut on Fig. 4B.

	Chr	Gene	Type	Ref.	Allele	Coding region change in longest transcript	Region	Start of Treatment			End of Treatment		
								Coverage	Freq	Avg quality	Coverage	Freq	Avg quality
CD4+ T-Cells	2	IDH1	SNV	T	A	NM_001282387.1:c.745A>T	209106823	0	0	n.a	411	7.1	39.5
	2	BARD1	SNV	C	G	NM_000465.3:c.1603G>C	215617245	0	0	n.a	5821	86.2	45.8
	3	FANCD2	SNV	T	G	NM_033084.4:c.1156T>G	10088285	1776	22.8	51.1	2089	5.8	54.9
	3	FANCD2	SNV	G	T	NM_033084.4:c.2444G>T	10108951	2337	4.2	47.3	0	0	n.a
	3	ATR	SNV	G	C	NM_001184.3:c.7382C>G	142177921	0	0	n.a	882	4.6	51.9
	3	ATR	Del	TC	-	NM_001184.3:c.3081_3082delGA	142268410..142268411	1971	5.9	49.3	0	0	n.a
	4	FAM175A	SNV	G	T	NM_139076.2:c.387C>A	84391445	3194	2.1	47.2	671	4.2	48.8
	9	FANCC	SNV	G	A	NM_000136.2:c.1036C>T	97879633	0	0	n.a	346	13.9	50.0
	9	FANCC	Ins	-	T	NM_000136.2:c.265dupA	98003010^98003011	0	0	n.a	397	15.4	50.6
	11	ATM	Ins	-	A	NM_000051.3:c.4741dupA	108164163^108164164	0	0	n.a	601	5.2	56.5
	11	ATM, C11orf65	SNV	T	A	NM_000051.3:c.7113T>A	108199771	1312	4.2	48.5	0	0	n.a
	11	ATM, C11orf65	SNV	T	G	NM_000051.3:c.7116T>G	108199774	1348	4.3	48.5	0	0	n.a
	11	ATM, C11orf65	Ins	-	C	NM_000051.3:c.7660dupC	108202631^108202632	0	0	n.a	379	12.1	50.3
	13	BRCA2	SNV	G	T	NM_000059.3:c.3599G>T	32912091	0	0	n.a	1120	4.7	48.0
	13	BRCA2	SNV	G	A	NM_000059.3:c.3919G>A	32912411	0	0	n.a	1112	13.8	46.5
	13	BRCA2	SNV	A	C	NM_000059.3:c.7640A>C	32931901	0	0	n.a	513	8.4	47.4
	13	BRCA2	SNV	C	G	NM_000059.3:c.7642C>G	32931903	0	0	n.a	512	8.0	47.8
	13	BRCA2	SNV	T	A	NM_000059.3:c.7644T>A	32931905	0	0	n.a	512	8.4	47.9
	13	BRCA2	Ins	-	T	NM_000059.3:c.7704dupT	32931961^32931962	0	0	n.a	1560	4.0	51.3
	13	BRCA2	SNV	C	G	NM_000059.3:c.8165C>G	32937504	2104	55.9	49.6	309	80.3	49.3
	15	IDH2	Ins	-	C	NM_002168.3:c.435dupG	90631917^90631918	4646	7.0	47.3	1031	11.2	50.0
	17	RAD51L3-RFFL, RAD51D	SNV	T	C	NM_001142571.1:c.269A>G	33443932	632	75.2	47.7	0	0	n.a
	17	CDK12	Ins	-	G	NM_016507.3:c.4382dupG	37687471^37687472	2161	11.0	45.4	712	7.2	53.0
	17	BRCA1	SNV	G	A	NM_007300.3:c.2765C>T	41244783	0	0	n.a	735	8.3	49.0
	17	BRCA1	SNV	T	G	NM_007300.3:c.953A>C	41246595	0	0	n.a	704	7.8	48.6
	17	BRIP1	Ins	-	AT	NM_032043.2:c.2571_2572dupAT	59770793^59770794	0	0	n.a	1293	8.0	52.9
	17	RAD51C	SNV	C	T	NM_058216.2:c.277C>T	56772423	0	0	n.a	497	5.2	49.1
	22	CHEK2	SNV	C	T	NM_001005735.1:c.1733G>A	29083913	0	0	n.a	285	6.0	36.6

Table S15. NGS results from the sequencing of patient 02 buffy coat. Colored genes match donuts in **Figure 4B**. Genes were colored to match **Figure 4B** only if the region matched exactly. If variant also found in buffy coat, it is stated and colored green. "NEAR region" is the region of target gene sequenced from isolated CTCs. "Detected cell type" shows cell type, collection date, and frequency for the given gene variant. See **Tables S11-S14** for full CTC sequencing data.

Sequencing data of buffy coat for patient 02										Compared to CTC sequencing			
Chr	Gene	Type	Ref.	Allele	Coverage	Freq.	Avg quality	Coding region change in longest transcript	Region	**NEAR** Region	Coding region change in longest transcript	Detected cell type	
13	BRCA2	Ins.	-	A	3747	40.19215372	45.0062	ENST0000054 4455:c.1813du pA	32907420^329 07421	32907420^3 2907421	NM_000059.3:c.1 813dupA	buffy	EpCAM C1 (36.3 freq); FAP C1 (36.3 freq); CD4 C1 (28.4 freq)
17	CDK12	Del.	TCC	-	6459	50.13159932	44.5297	ENST0000044 7079:c.3770_3 772delCTC	37686861..376 86863	37686861.. 37686863	NM_016507.3:c.3 770_3772delCT C	buffy	EpCAM C1 (37.1 freq); EpCAM EOT(98.5 freq); FAP C1 (47.3 freq); FAP EOT (97.2 freq); CD4 C1(36.2 freq); CD4 EOT (2.2 freq)

Table S16. NGS results from the sequencing of patient 02 baseline cfDNA. Colored genes match donuts in **Figure 4B**. Genes were colored to match **Figure 4B** only if the region matched exactly. If variant also found in buffy coat, it is stated and colored green. "NEAR region" is the region of target gene sequenced from isolated CTCs. Some gene variants were detected in CTCs within a 10,000 nucleotide region from those detected in cfDNA and were included here. "Detected cell type" shows cell type, collection date, and frequency for the given gene variant. See **Tables S11-S14** for full CTC sequencing data.

Sequencing data of baseline cfDNA patient 02										Compared to CTC sequencing			
Chr	Gene	Type	Ref.	Allele	Coverage	Freq.	Avg quality	Coding region change in longest transcript	Region	**NEAR** Region	Coding region change in longest transcript	Detected cell type	
13	BRCA2	Ins.	-	A	382	40.05235602	52.8083	ENST0000054 4455:c.1813du pA	32907420^329 07421	32907420^3 2907421	NM_000059.3:c.1 813dupA	buffy	EpCAM C1 (36.3 freq); FAP C1 (36.3 freq); CD4 C1 (28.4 freq)
17	CDK12	Del.	TCC	-	1307	54.5524101	50.0492	ENST0000044 7079:c.3770_3 772delCTC	37686861..376 86863	37686861.. 37686863	NM_016507.3:c.3 770_3772delCT C	buffy	EpCAM C1 (37.1 freq); EpCAM EOT(98.5 freq); FAP C1 (47.3 freq); FAP EOT (97.2 freq); CD4 C1(36.2 freq); CD4 EOT (2.2 freq)
17	BRIP1	Del.	TTTG	-	756	1.058201058	39	ENST0000025 9008:c.2990_2 993delCAAA	59761414..597 61417	59760726	NM_032043.2:c.3 681T>A	FAP EOT, (4.6 freq)	

Table S17. NGS results from the sequencing of patient 02 end-of-treatment (EOT) cfDNA. Colored genes match donuts in **Figure 4B**. Genes were colored to match **Figure 4B** only if the region matched exactly. If variant also found in buffy coat, it is stated and colored green. "NEAR region" is the region of target gene sequenced from isolated CTCs. Some gene variants were detected in CTCs within a 10,000 nucleotide region from those detected in cfDNA and were included here. "Detected cell type" shows cell type, collection date, and frequency for the given gene variant. See **Tables S11-S14** for full CTC sequencing data.

Sequencing data of EOT cfDNA patient 02										Compared to CTC sequencing			
Chr	Gene	Type	Ref.	Allele	Coverage	Freq.	Avg quality	Coding region change in longest transcript	Region	**NEAR** Region	Coding region change in longest transcript	Detected cell type	
3	FANCD2	SNV	C	T	314	1.592356688	56.2	ENST00000287647:c.3931C>T	10136015	10132010	NM_033084.4:c.3718G>A	EpCAM EOT (2.9 freq)	
13	BRCA2	Ins.	-	A	243	40.32921811	52.9286	ENST00000544455:c.1813dupA	32907420^32907421	32907420^32907421	NM_000059.3:c.1813dupA	buffy	EpCAM C1 (36.3 freq); FAP C1 (36.3 freq); CD4 C1 (28.4 freq)
13	BRCA2	MNV	CT	TC	146	1.369863014	44.25	ENST00000544455:c.8361_8362delinsTC	32944568..32944569	32944572	NM_000059.3:c.8365T>A	CD4 EOT (2.7 freq)	
17	CDK12	Del.	TCC	-	928	49.89224138	54.0014	ENST00000447079:c.3770_3772delCTC	37686861..37686863	37686861..37686863	NM_016507.3:c.3770_3772delCTC	buffy	EpCAM C1 (37.1 freq); EpCAM EOT(98.5 freq); FAP C1 (47.3 freq); FAP EOT (97.2 freq); CD4 C1(36.2 freq); CD4 EOT (2.2 freq)

Table S18. NGS results from the sequencing of patient 06 buffycoat. Colored genes match donuts in **Figure 4B**. Genes were colored to match **Figure 4B** only if the region matched exactly. If variant also found in buffy coat, it is stated and colored green. "NEAR region" is the region of target gene sequenced from isolated CTCs. Some gene variants were detected in CTCs within a 10,000 nucleotide region from those detected in cfDNA and were included here. "Detected cell type" shows cell type, collection date, and frequency for the given gene variant. See **Tables S11-S14** for full CTC sequencing data.

Sequencing data of buffy coat for patient 06										Compared to CTC sequencing			
Chr	Gene	Type	Ref.	Allele	Coverage	Freq.	Avg quality	Coding region change in longest transcript	Region	**NEAR** Region	Coding region change in longest transcript	Detected cell type	
2	BARD1	MNV	CA	TG	4368	51.0989011	49.8243	ENST00000260947:c.1518_1519delinsCA	215632255..215632256	215617245	NM_000465.3:c.1603G>C	buffy	EpCAM EOT (98.0 freq); FAP EOT (98.7 freq); CD4 EOT (86.2 freq)
13	BRCA2	SNV	C	G	1498	51.26835781	51.6328	ENST00000544455:c.8165C>G	32937504	32937504	NM_000059.3:c.8165C>G	buffy	EpCAM C1 (46.3 freq); EpCAM EOT (n.d.); FAP C1 (34.1 freq); FAP EOT (99.1 freq) CD4 C1 (55.9 freq); CD4 EOT (80.3 freq)

Table S19. NGS results from the sequencing of patient 06 baseline cfDNA. Colored genes match donuts in **Figure 4B**. Genes were colored to match **Figure 4B** only if the region matched exactly. If variant also found in buffy coat, it is stated and colored green. "NEAR region" is the region of target gene sequenced from isolated CTCs. Some gene variants were detected in CTCs within a 10,000 nucleotide region from those detected in cfDNA and were included here. "Detected cell type" shows cell type, collection date, and frequency for the given gene variant. See **Tables S11-S14** for full CTC sequencing data. "n.d." means there was no variant within 10,000 nucleotides detected in CTCs.

Sequencing data of baseline cfDNA for patient 06										Compared to CTC sequencing			
Chr	Gene	Type	Ref.	Allele	Coverage	Freq.	Avg quality	Coding region change in longest transcript	Region	**NEAR** Region	Coding region change in longest transcript	Detected cell type	
1	RAD54L	SNV	A	C	175	1.714285714	44.6667	ENST00000371975:c.197A>C	46715778	n.d.	n.d.	n.d.	n.d.
1	RAD54L	SNV	A	T	302	1.324503311	48	ENST00000371975:c.2093A>T	46743803	n.d.	n.d.	n.d.	n.d.
2	GEN1	MNV	CA	GG	133	3.007518797	38.625	ENST00000381254:c.2655_2656delinsGG	17963134..17963135	n.d.	n.d.	n.d.	n.d.
2	BARD1	MNV	CA	TG	461	42.29934924	54.7327	ENST00000260947:c.1518_1519delinsCA	215632255..215632256	215617245	NM_000465.3:c.1603G>C	buffy	EpCAM EOT (98.0 freq); FAP EOT (98.7 freq); CD4 EOT (86.2 freq)
2	BARD1	SNV	G	C	102	1.960784314	60	ENST00000260947:c.70C>G	215674224	215610566	NM_000465.3:c.1690C>T	FAP C1 (5.0 freq)	
3	FANCD2	SNV	G	C	280	1.071428571	44	ENST00000287647:c.602G>C	10081436	10088285	NM_033084.4:c.1156T>G	EpCAM C1 (7.6 freq); FAP C1 (13.2 freq); CD4 C1 (22.1 freq); CD4 EOT (5.8 freq)	
9	FANCG	SNV	A	T	131	3.816793893	45.6	ENST00000378643:c.1721T>A	35074407	n.d.	n.d.	n.d.	n.d.
17	BRCA1	Del.	C	-	152	1.973684211	42.6667	ENST00000309486:c.4557delG	41199682	n.d.	n.d.	n.d.	n.d.
17	BRIP1	Del.	TTTG	-	241	2.074688797	45.6	ENST00000259008:c.2990_2993delCAAA	59761414..59761417	n.d.	n.d.	n.d.	n.d.
22	CHEK2	SNV	C	G	136	1.470588235	60	ENST00000416671:c.526G>C	29121031	n.d.	n.d.	n.d.	n.d.

Table S20. NGS results from the sequencing of patient 06 end-of-treatment (EOT) cfDNA. Colored genes match donuts in **Figure 4B**. Genes were colored to match **Figure 4B** only if the region matched exactly. If variant also found in buffy coat, it is stated and colored green. "NEAR region" is the region of target gene sequenced from isolated CTCs. Some gene variants were detected in CTCs within a 10,000 nucleotide region from those detected in cfDNA and were included here. "Detected cell type" shows cell type, collection date, and frequency for the given gene variant. See **Tables S11-S14** for full CTC sequencing data. "n.d." means there was no variant within 10,000 nucleotides detected in CTCs.

Sequencing data of EOT cfDNA for patient 06										Compared to CTC sequencing			
Chr	Gene	Type	Ref.	Allele	Coverage	Freq.	Avg quality	Coding region change in longest transcript	Region	**NEAR** Region	Coding region change in longest transcript	Detected cell type	
2	BARD1	MNV	CA	TG	624	51.44230769	53.5359	ENST00000260947:c.1518_1519delinsCA	215632255..215632256	215617245	NM_000465.3:c.1603G>C	buffy	EpCAM EOT (98.0 freq); FAP EOT (98.7 freq); CD4 EOT (86.2 freq)
3	BAP1	SNV	C	T	269	2.230483271	39.3333	ENST00000460680:c.2159G>A	52436335	n.d.	n.d.	n.d.	n.d.
3	ATR	SNV	T	G	322	1.863354037	36.6667	ENST00000350721:c.2149A>C	142274911	142268410..142268411	NM_001184.3:c.3081_3082delGA		CD4 C1 (5.9 freq)
3	ATR	MNV	CA	TT	116	2.586206897	50.4447	ENST00000350721:c.1519_1520delinsAA	142279126..142279127	142281719	NM_001184.3:c.525G>A		EpCAM EOT (5.1 freq)
8	NBN	Del.	CTG	-	239	1.255230126	36	ENST00000396252:c.*1224_*1226delCAG	90967555..90967557	90967511^90967512	NM_001024688.2:c.1150dupA		FAP EOT (6.1 freq)
13	BRCA2	SNV	C	G	104	54.80769231	55.1053	ENST00000544455:c.8165C>G	32937504	32937504	NM_000059.3:c.8165C>G	buffy	EpCAM C1 (46.3 freq); EpCAM EOT (n.d.); FAP C1 (34.1 freq); FAP EOT (99.1 freq) CD4 C1 (55.9 freq); CD4 EOT (80.3 freq)
14	RAD51B	SNV	C	T	239	2.510460251	50.6667	ENST00000487270:c.784C>T	68758628	n.d.	n.d.	n.d.	n.d.

Table S21. NGS results from the sequencing of patient 06 healthy tissue. Colored genes match donuts in **Figure 4B**. Genes were colored to match **Figure 4B** only if the region matched exactly. If variant also found in buffy coat, it is stated and colored green. "NEAR region" is the region of target gene sequenced from isolated CTCs. Some gene variants were detected in CTCs within a 10,000 nucleotide region from those detected in cfDNA and were included here. "Detected cell type" shows cell type, collection date, and frequency for the given gene variant. See **Tables S11-S14** for full CTC sequencing data. "n.d." means there was no variant within 10,000 nucleotides detected in CTCs.

Sequencing data of healthy tissue for patient 06										Compared to CTC sequencing			
Chr	Gene	Type	Ref.	Allele	Coverage	Freq.	Avg quality	Coding region change in longest transcript	Region	**NEAR** Region	Coding region change in longest transcript	Detected cell type	
2	BARD1	MNV	CA	TG	271	46.49446494	53.9919	ENST00000260947:c.1518_1519delinsCA	215632255..215632256	215617245	NM_000465.3:c.1603G>C	buffy	EpCAM EOT (98.0 freq); FAP EOT (98.7 freq); CD4 EOT (86.2 freq)
3	ATR	SNV	G	A	159	6.918238994	40.5455	ENST00000350721:c.2821C>T	142269129	142268410..142268411	NM_001184.3:c.3081_3082delGA		CD4 C1 (5.9 freq)
13	BRCA2	SNV	T	C	125	4	45.6	ENST00000544455:c.9715T>C	32972365	n.d.	n.d.	n.d.	n.d.
16	PALB2	SNV	C	T	146	4.794520548	46.2857	ENST00000261584:c.2039G>A	23641436	n.d.	n.d.	n.d.	n.d.
17	RPA1	SNV	C	T	342	5.263157895	39.3333	ENST00000254719:c.521C>T	1779021	n.d.	n.d.	n.d.	n.d.
17	BRIP1	SNV	G	T	153	5.882352941	54.2222	ENST00000259008:c.2180C>A	59821870	59770793^59770794	NM_032043.2:c.2571_2572dupAT		CD4 EOT (8.0 freq)

Table S22. NGS results from the sequencing of patient 06 solid tumor tissue. Colored genes match donuts in **Figure 4B**. Genes were colored to match **Figure 4B** only if the region matched exactly. If variant also found in buffy coat, it is stated and colored green. "NEAR region" is the region of target gene sequenced from isolated CTCs. Some gene variants were detected in CTCs within a 10,000 nucleotide region from those detected in cfDNA and were included here. "Detected cell type" shows cell type, collection date, and frequency for the given gene variant. See **Tables S11-S14** for full CTC sequencing data. "n.d." means there was no variant within 10,000 nucleotides detected in CTCs.

Sequencing data of tumor tissue for patient 06										Compared to CTC sequencing			
Chr	Gene	Type	Ref.	Allele	Coverage	Freq.	Avg quality	Coding region change in longest transcript	Region	**NEAR** Region	Coding region change in longest transcript	Detected cell type	
1	RAD54L	SNV	C	T	288	4.513888889	48.6923	ENST00000371975:c.1031C>T	46733270	n.d.	n.d.	n.d.	n.d.
2	BARD1	MNV	CA	TG	622	47.26688103	53.1168	ENST00000260947:c.1518_1519delinsCA	215632255..215632256	215617245	NM_000465.3:c.1603G>C	buffy	EpCAM EOT (98.0 freq); FAP EOT (98.7 freq); CD4 EOT (86.2 freq)
9	FANCG	SNV	G	A	187	4.812834225	48	ENST00000378643:c.1162C>T	35075733	n.d.	n.d.	n.d.	n.d.
9	FANCG	SNV	G	A	170	4.117647059	43.4286	ENST00000378643:c.1157C>T	35075738	n.d.	n.d.	n.d.	n.d.
9	FANCG	SNV	G	A	130	4.615384615	43.8333	ENST00000378643:c.1150C>T	35075745	n.d.	n.d.	n.d.	n.d.
11	ATM	SNV	G	A	122	8.196721311	38.8	ENST00000278616:c.7876G>A	108203576	108202631^108202632	NM_000051.3:c.7660dupC	CD4 EOT (12.1 freq)	
13	BRCA2	SNV	G	A	177	5.649717514	39.1	ENST00000544455:c.7288G>A	32929278	32929161^32929162	NM_000059.3:c.7177dupA	EpCAM C1 (1.0 freq); EpCAM EOT (9.1 freq)	
13	BRCA2	SNV	T	A	146	5.479452055	37.875	ENST00000544455:c.7742T>A	32932003	32931905	NM_000059.3:c.7644T>A	CD4 EOT (8.4 freq)	
17	BRCA1	SNV	C	T	200	7.5	45.6	ENST00000309486:c.3983G>A	41223060	n.d.	n.d.	n.d.	n.d.
17	BRIP1	SNV	C	T	182	5.494505495	45.4	ENST00000259008:c.2026G>A	59853833	59770793^59770794	NM_032043.2:c.2571_2572dupAT	CD4 EOT (8.0 freq)	

Table S23. LDR cell-line control experiments. LDR was performed on gDNA extracted from positive control cell lines known to have KRAS mutation (RPMI 8226 (multiple myeloma cell line), HCT 116 (colon cancer cell line), SW480 (colorectal cancer cell line), and negative control cell lines; HT-29 (wild type (WT), adenocarcinoma colorectal cancer cell line), SKBR3 (breast cancer cell line). (+) is indicative of mutations that were detected using LDR that were expected. (–) symbol means that the mutation was not detected and not expected.

Cell line	WT (G35G)	G35T	G35A	G35C	WT (G34G)	G34C
RPMI 8226	+	+	-	-	+	-
HCT116	+	-	-	-	+	-
HT-29	+	-	-	-	+	-
SKBR3	+	-	-	-	+	-
SW480	+	-	+	+	+	-

Table S24. Ligase Detection Reaction primers sequences and lengths.

KRAS Mutation	Discriminating primer 5'-3 (nt)	Common primer 5'-3' (nt)	Ligated product size
**GGTGGC 35 WT**	TTTTTTTAAACTTGTGGTAGTTGGA GCTGG (30 nt)	PhTGG CGTAGGCAAGAGTGCCT-Cy5 (20 nt)	**50 nt**
**GATGGC G35A**	TAAACTTGTGGTAGTTGGAGCTGA (24 nt)	PhTGGCGTAGGCAAGAGTGCCT-Cy5 (20 nt)	**44 nt**

**GTTGGC G35T**	TTTTTTTTTTTTTTAAACTTGTGGTA GTTGGAGCTGT (35 nt)	PhTGGCGTAGGCAAGAGTGCCT-Cy5 (20 nt)	**55 nt**
**GGTGGC 34 WT**	TTTTTTTTTTTTTTTTAAACTTGTGGT AGTTGGAGCTG (37 nt)	PhGTGGCGTAGGCAAGAGTGCCTTGA CGATAC-Cy5 (30 nt)	**67 nt**
**CGTGGC G34C**	TTTTTTTTTAAACTTGTGGTAGTTG GAGCTC (31 nt)	PhGTGGCGTAGGCAAGAGTGCCTTGA CGATAC-Cy5 (30 nt)	**61 nt**

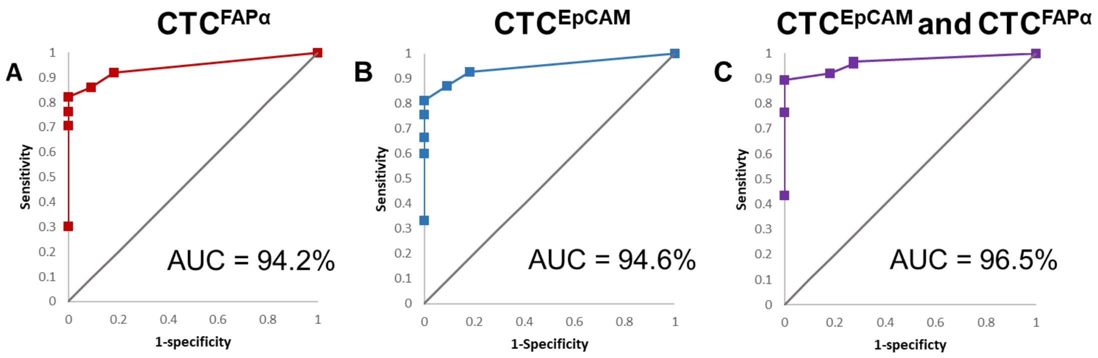

Figure S1. ROC analysis for both subpopulations of CTCs. Area under the curve (AUC) for different CTC subpopulations for determining minimal residual PDAC disease in metastatic patients.

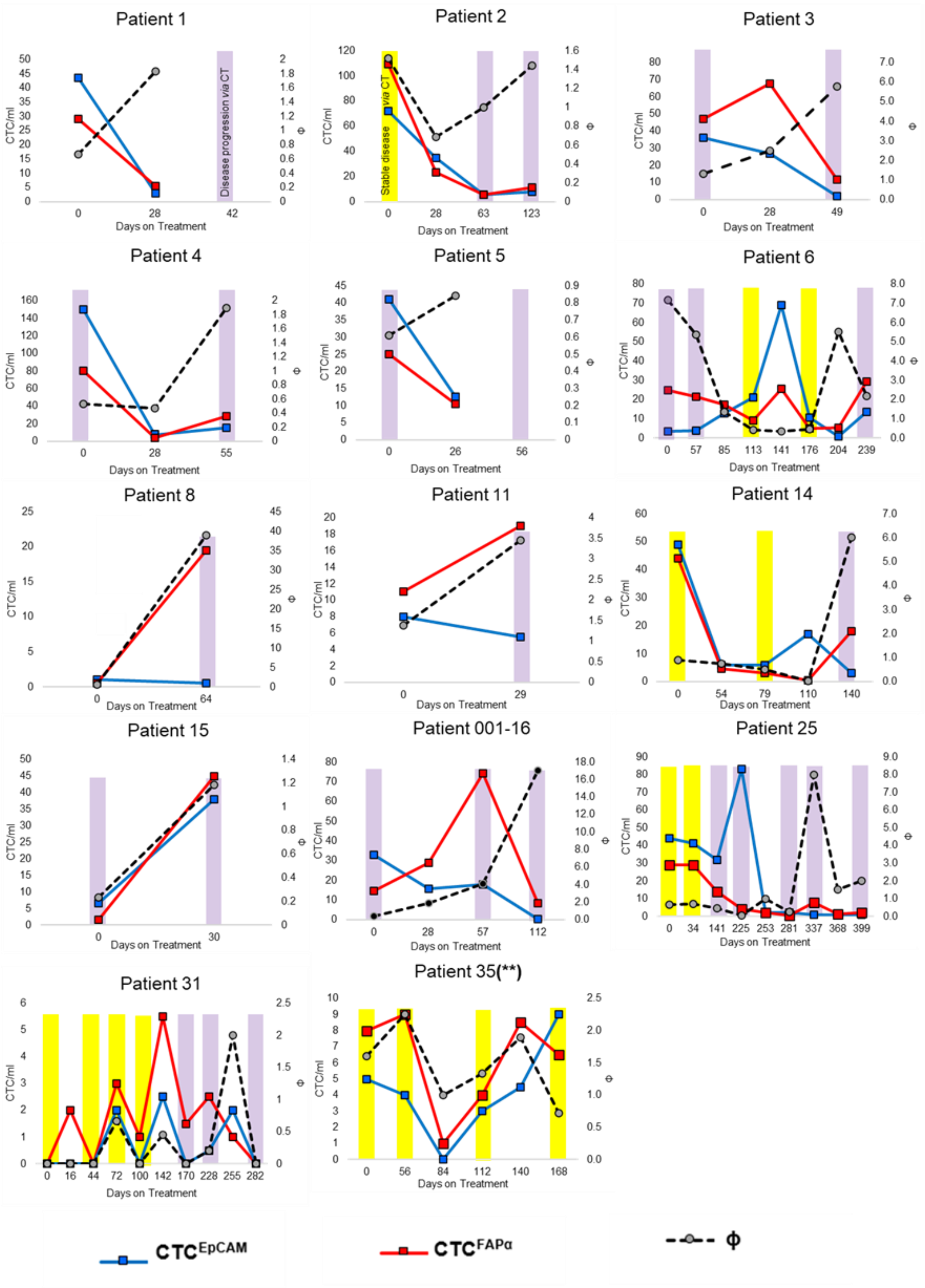

Figure S2. Φ & CTC^{EpCAM} and CTC$^{FAP\alpha}$ for patients in which data show correlation of ΔΦ with disease status *via* CT. Purple bars overlaid on individual patient graphs represent disease progression determined *via* CT; yellow bars represent stable disease determined *via* CT by clinicians. Patients with colored bars at day 0 of treatment shows difference in progression (if any) from baseline scan.

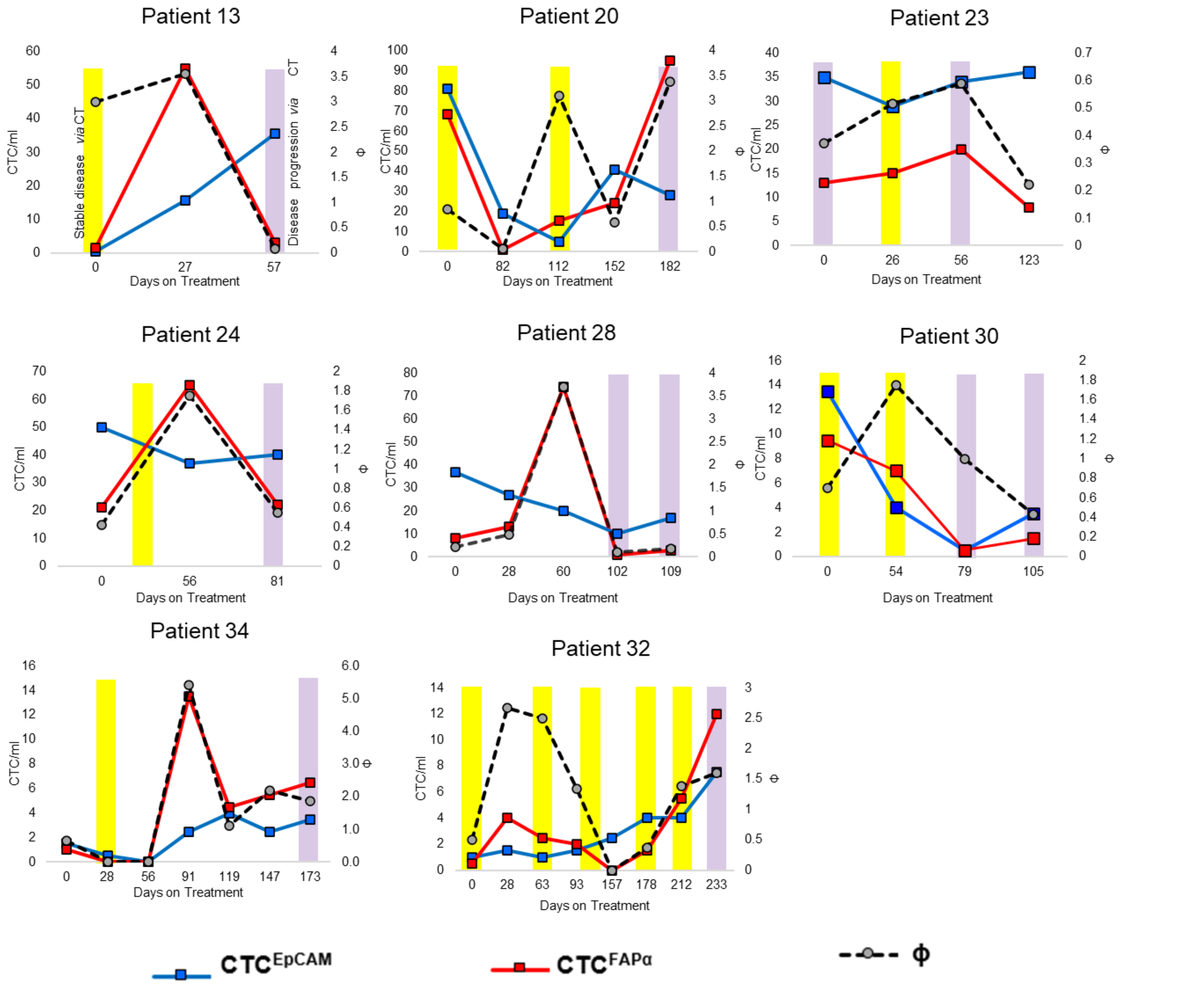

Figure S3. Φ & CTC^{EpCAM} and $CTC^{FAP\alpha}$ for patients in which data show correlation along with disease status via CT, with modulation observed. Purple bars overlaid on individual patient graphs represent disease progression determined *via* CT; yellow bars represent stable disease determined *via* CT by clinicians. Patients with colored bars at day 0 of treatment shows difference in progression (if any) from baseline scan. Patients 33 & 35 are still enrolled in therapy at time of data presentation and are marked with (**).

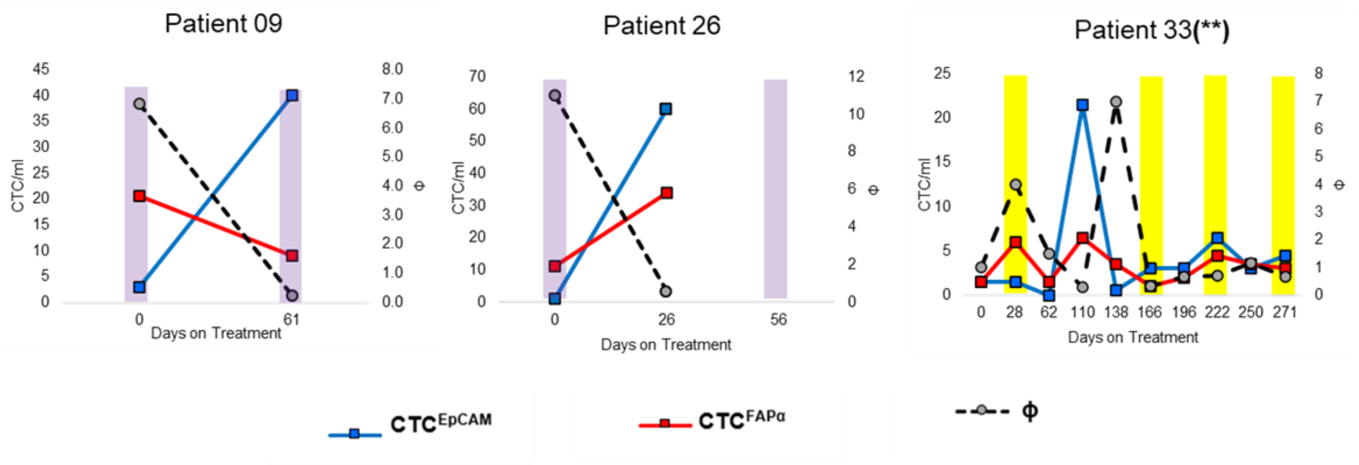

Figure S4. Φ & CTC^EpCAM^ and CTC^FAPα^ for patients in which data show no correlation to CT. Purple bars overlaid on individual patient graphs represent disease progression determined *via* CT; yellow bars represent stable disease determined *via* CT by clinicians. Patients with colored bars at day 0 of treatment shows difference in progression (if any) from baseline scan.

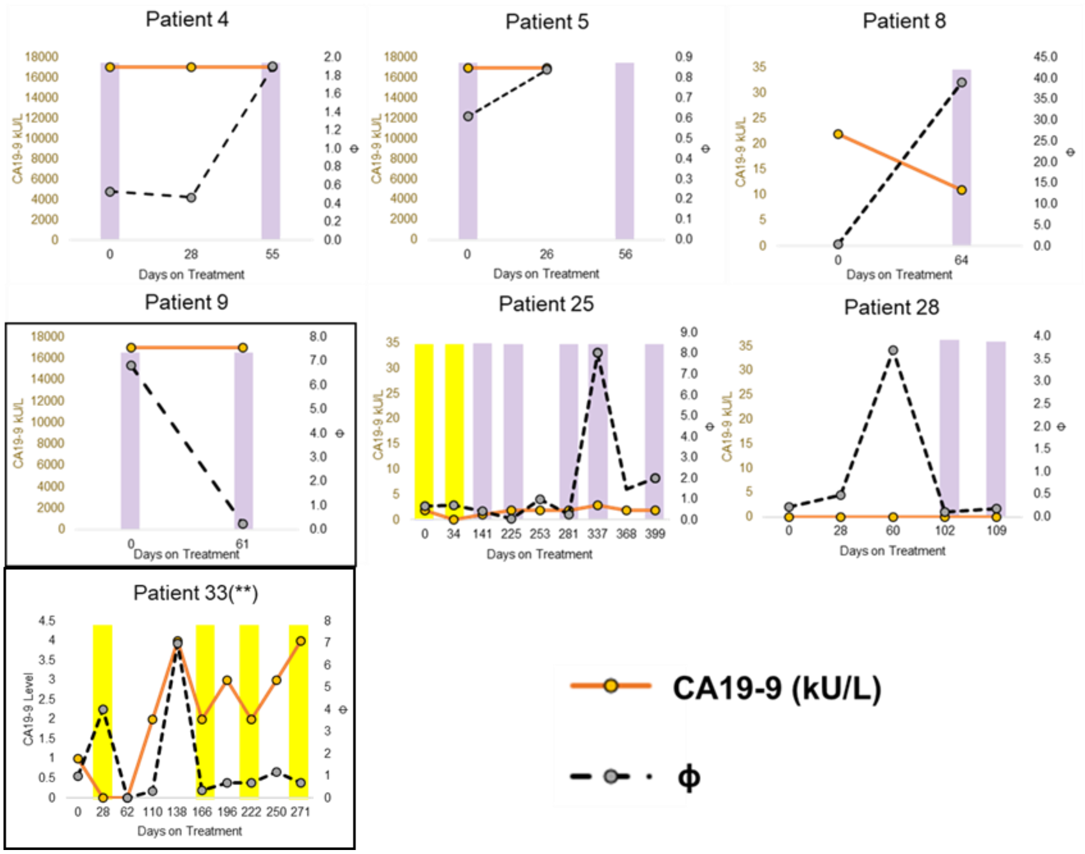

Figure S5. Patients for whom CA19-9 was immeasurable and in 5/7 cases Φ shows advantage over CA19-9. Graphs in the boxes show for pt#9 and pt#3 show no advantage of Φ. Purple bars overlaid on individual patient graphs represent disease progression determined *via* CT; yellow bars represent stable disease determined *via* CT by clinicians. Patients with colored bars at day 0 of treatment shows difference in progression (if any) from baseline scan.

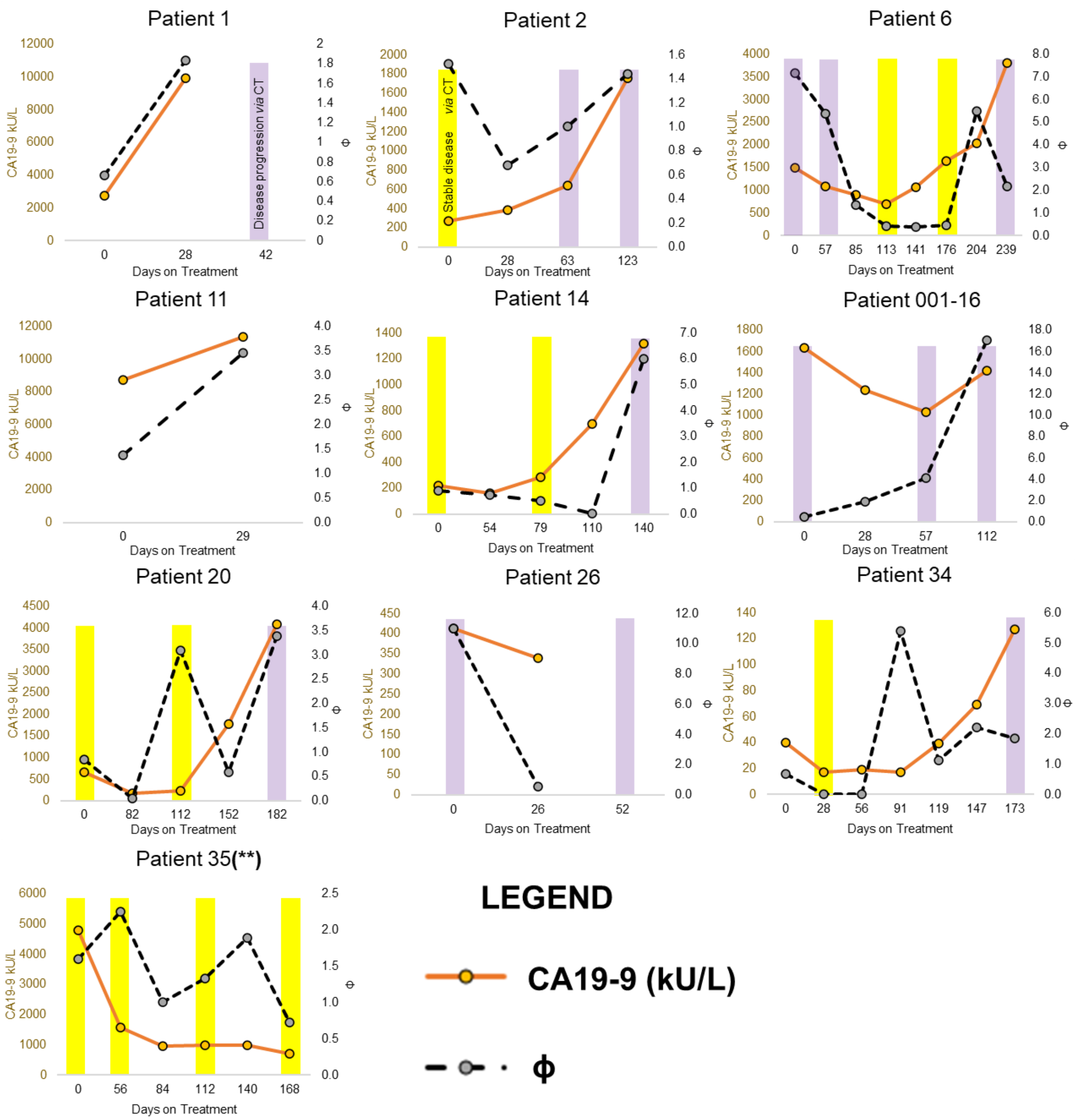

Figure S6. Patients for which a positive correlation between Φ & CA19-9, and disease status via CT. Purple bars overlaid on individual patient graphs represent disease progression determined *via* CT; yellow bars represent stable disease determined *via* CT by clinicians. Patients with colored bars at day 0 of treatment shows difference in progression (if any) from baseline scan.

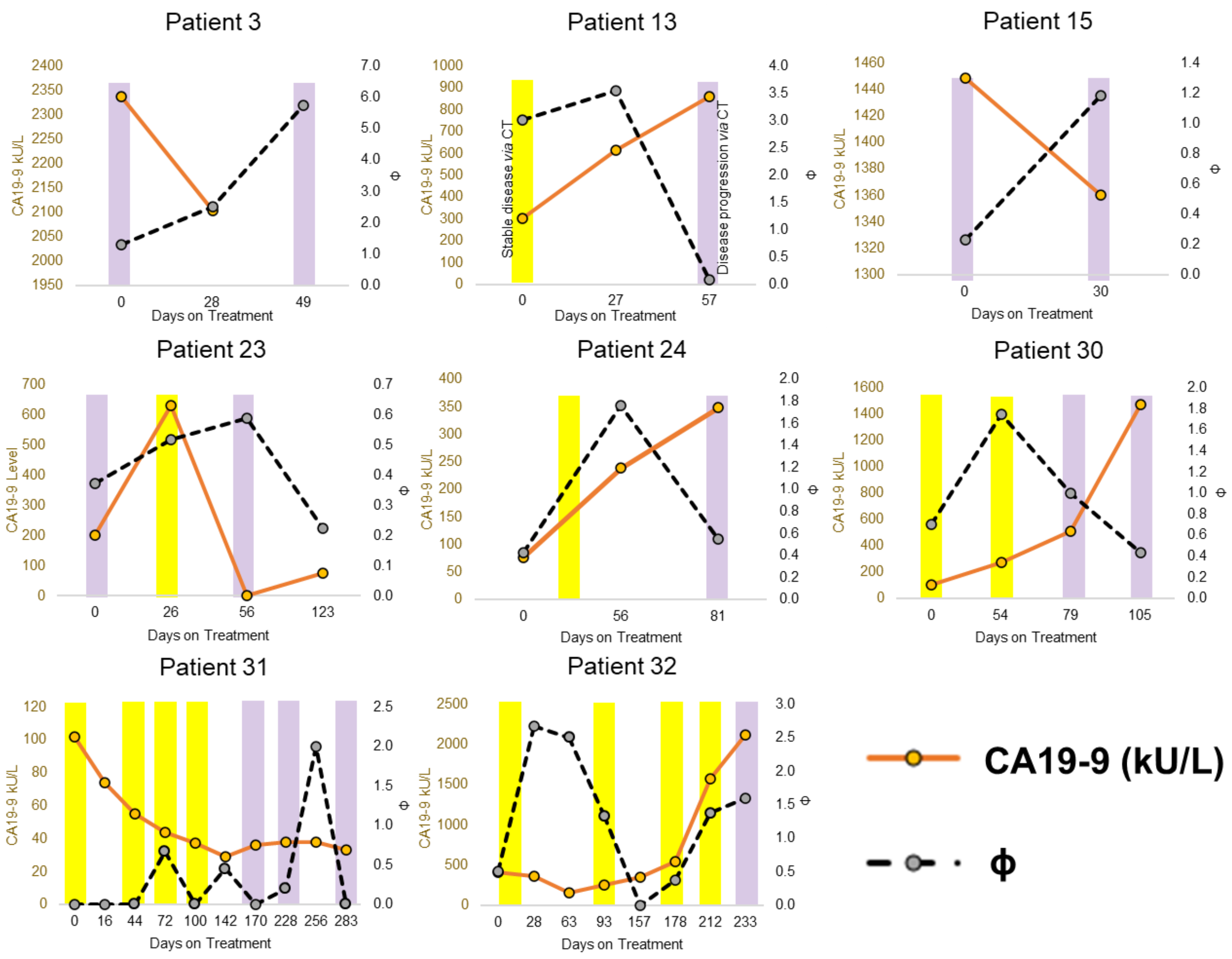

Figure S7. Patients for which no apparent correlation between Φ & CA19-9 is observed, and disease status via CT. Purple bars overlaid on individual patient graphs represent disease progression determined *via* CT; yellow bars represent stable disease determined *via* CT by clinicians. Patients with colored bars at day 0 of treatment shows difference in progression (if any) from baseline scan.

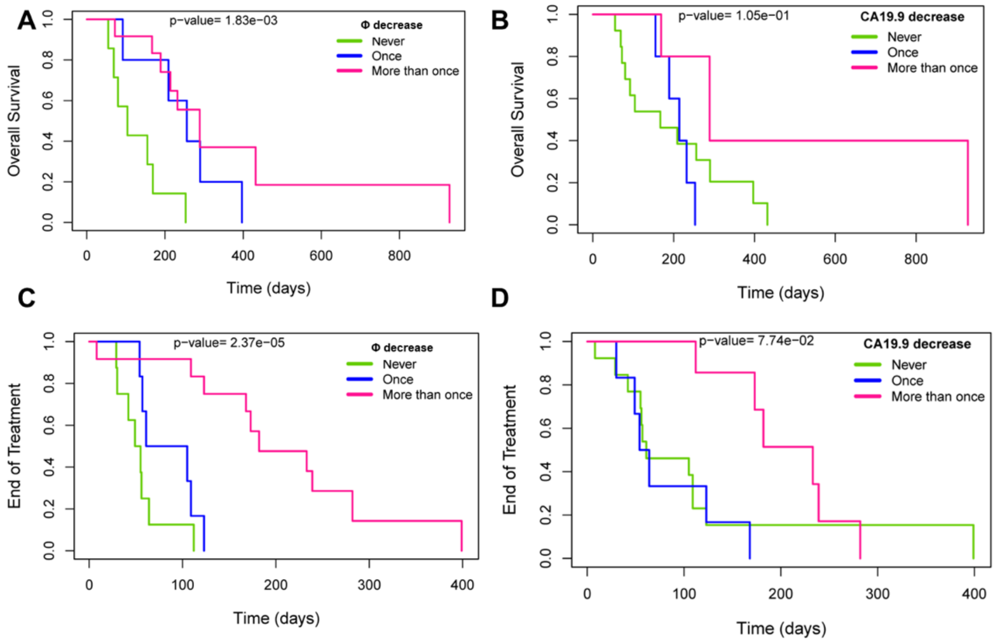

Figure S8. Kaplan-Meier plots for overall survival (OS) and end of treatment (EOT). PDAC patients using (**A**) ΔΦ (n=26) and (**B**) ΔCA19-9 (n=26). Prediction of EOT (*i.e.*, progression free survival) for PDAC patients using (**C**) ΔΦ (n=26) and (**D**) ΔCA19-9 (n=26). *T*hree groups of patients were evaluated; those for whom no decrease in Φ (n=8) and separately CA19-9 marker decrease, (n=13) was observed, those for whom Φ and CA19-9 decreased only one time (n=6 and n=7, respectively), and the third group of patients, for whom Φ (n=12) and CA19-9 (n=6) decreased more than one time. .

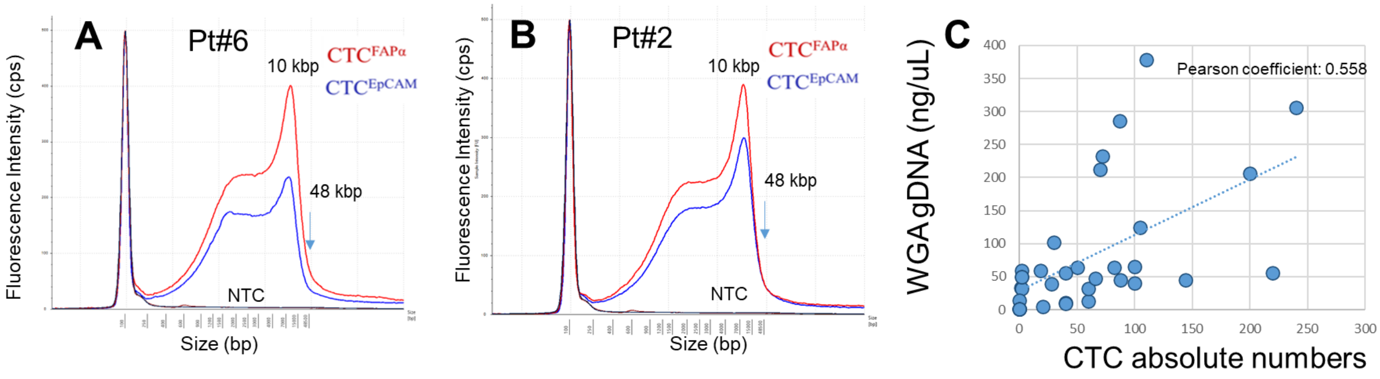

Figure S9. Electrophoretic traces of WGA gDNA. (A) gDNA extracted from CTC isolated from blood of Pt#06 and (B) Pt#02, and WGA. The mode of WGA amplicons is ~10kbp. (C) Correlation between number of CTC isolated and yield of WGA products. Pearson correlation shows positive correlation.
